# Molecular identification of *Baylisascaris melis* (Gedoelst, 1920) from the Eurasian badger (*Meles meles*) and ascarids from other wild carnivores in Kazakhstan

**DOI:** 10.3389/fvets.2024.1452237

**Published:** 2024-09-09

**Authors:** Rabiga Uakhit, Ainura Smagulova, Lyudmila Lider, Alexandr Shevtsov, Alexandr A. Berber, Alexandr P. Berber, Christian Bauer, Vladimir Kiyan

**Affiliations:** ^1^Laboratory of Biodiversity and Genetic Resources, National Center for Biotechnology, Astana, Kazakhstan; ^2^Laboratory of Parasitology, Department of Veterinary Medicine, S. Seifullin Kazakh Agrotechnical Research University, Astana, Kazakhstan; ^3^Consortium of Hunting, Tourist and Fishing Farms "Adal Zher", Temirtau, Kazakhstan; ^4^Institute of Parasitology, Justus Liebig University Giessen, Giessen, Germany

**Keywords:** *Baylisascaris melis*, *Toxascaris leonina*, *Toxocara cati*, wild carnivores, mustelids, molecular identification, phylogeny, Kazakhstan

## Abstract

**Introduction:**

The presence of gastrointestinal nematodes, including zoonotic ascarids, in wild canids, felids and mustelids as definitive hosts in Central Asian countries has been documented in many studies based on traditional morphological methods. In contrast, relevant data for the badger are scarce. The aim of this study was the molecular identification of ascarid nematodes from five wild carnivore species in different regions of Kazakhstan.

**Methods:**

A total of 211 adult ascarids were collected from gray wolves (*Canis lupus*, 8 of 83 infected with 2–6 *Toxascaris leonina*), red foxes (*Vulpes vulpes*, 26 of 53, with 2–8 *Toxascaris leonina*), corsac foxes (*Vulpes corsac*, 6 of 11, 3–6 *Toxascaris leonina*), lynx (*Lynx lynx*, 2 of 3, with 2–5 *Toxocara cati*) and badgers (*Meles meles*, 2 of 4, with 2–7 *Baylisascaris melis*). Genomic DNA was extracted from the worms and ribosomal DNA, including the first and second internal transcribed spacer genes, was amplified by polymerase chain reaction using specific oligonucleotide primers and then sequenced.

**Results:**

*Toxascaris leonina*, but not *Toxocara canis*, was molecularly identified in the wild canids, *Toxocara cati* in the lynx and *Baylisascaris melis* in the badger. The maximum likelihood phylogenetic tree showed three distinct clades: the canid *Toxascaris leonina* was placed in one clade, *Toxocara cati* in another and *Baylisascaris melis* in a third.

**Discussion:**

The study provides the world’s first molecular data and phylogenetic analysis of *Baylisascaris melis*, identified for the second time since its description over 100 years ago. This species was shown to be genetically distinct from other *Baylisascaris* spp. (*B. columnaris*, *B. procyonis*, *B. transfuga*, *B. devosi*). The possible zoonotic significance of ascarids from wild carnivores is discussed in the light of conditions in Central Asia.

## 1 Introduction

Members of the genera *Toxocara*, *Toxascaris*, and *Baylisascaris* comprise the spectrum of ascarid nematodes (order Ascaridida: family Ascarididae) of terrestrial mammals, including the carnivores Canidae, Felidae, and Mustelidae (1, 2). Their adult stages parasitize the small intestines of the definitive host, which contaminates the environment by excreting worm eggs in feces. The eggs embryonate, can survive for months or years, and are ingested by another animal. Paratenic hosts (e.g., in *Toxocara* spp.) or intermediate hosts (in *Baylisascaris* spp.) may be facultatively involved, e.g., prey rodents. After oral ingestion of infective eggs, larvae penetrate the intestinal mucosa and migrate to the liver and other tissues, including the brain (3, 4). The infection can also be transmitted to humans (known as ‘toxocariasis’) (5). For example, the seroprevalence of toxocariasis in humans has been reported to be 11% in eastern Kazakhstan (6) and up to 54% in western Siberian regions of Russia (7). Depending on the ascarid species and the number of eggs ingested, the infection may be latent, but may also cause clinical symptoms (larva migrans syndrome) (4, 8). Contamination of the environment with ascarid eggs by domestic and wild carnivores is known in principle (4, 9, 10), but its impact in Central Asia is still unknown.

A number of studies have documented the occurrence and prevalence of helminth infections, including ascarids, in wild canids and felids in Kazakhstan [e.g., (11–14)] and neighboring countries [e.g., (15–19)]. In these studies, for example, wolves and red foxes were infected with *Toxocara canis* in 39% and 8–30% respectively, and with *Toxascaris* (*T*.) *leonina* in 38% and 6–78% respectively; *Toxocara cati* was present in 86% of lynx. In contrast, there are only two reports on the helminth fauna of badgers from Uzbekistan (17, 18), but no data from Kazakhstan. All these studies were carried out using traditional morphological methods. However, in field studies where the species identification of roundworms is based solely on their morphological features, the diagnosis is sometimes at least questionable, e.g., in badger (18–20). These diagnostic problems can be solved using molecular methods that have been available for many years. Such methods confirm or modify the taxonomic classification and can also be used to study the phylogenetic relationships of parasites such as ascarids, detect their genetic diversity and explain epidemiological results [e.g., (2, 21–24)]. Therefore, the aim of the present study was to molecularly confirm the morphological species diagnosis of roundworms from five wild carnivore species in different regions of Kazakhstan, including wolf, red fox, corsac fox, lynx and badger, and to provide baseline data for future investigations.

## 2 Materials and methods

### 2.1 Ethical approval

The study had been approved by the local Animal Ethics Committee (extract from Protocol No. 1 dated 24 July 2019) prior to commencement and was conducted in accordance with the World Medical Association Code of Ethics (Declaration of Helsinki) for animal research.^1^

### 2.2 Sample collection

**Figure 1:**
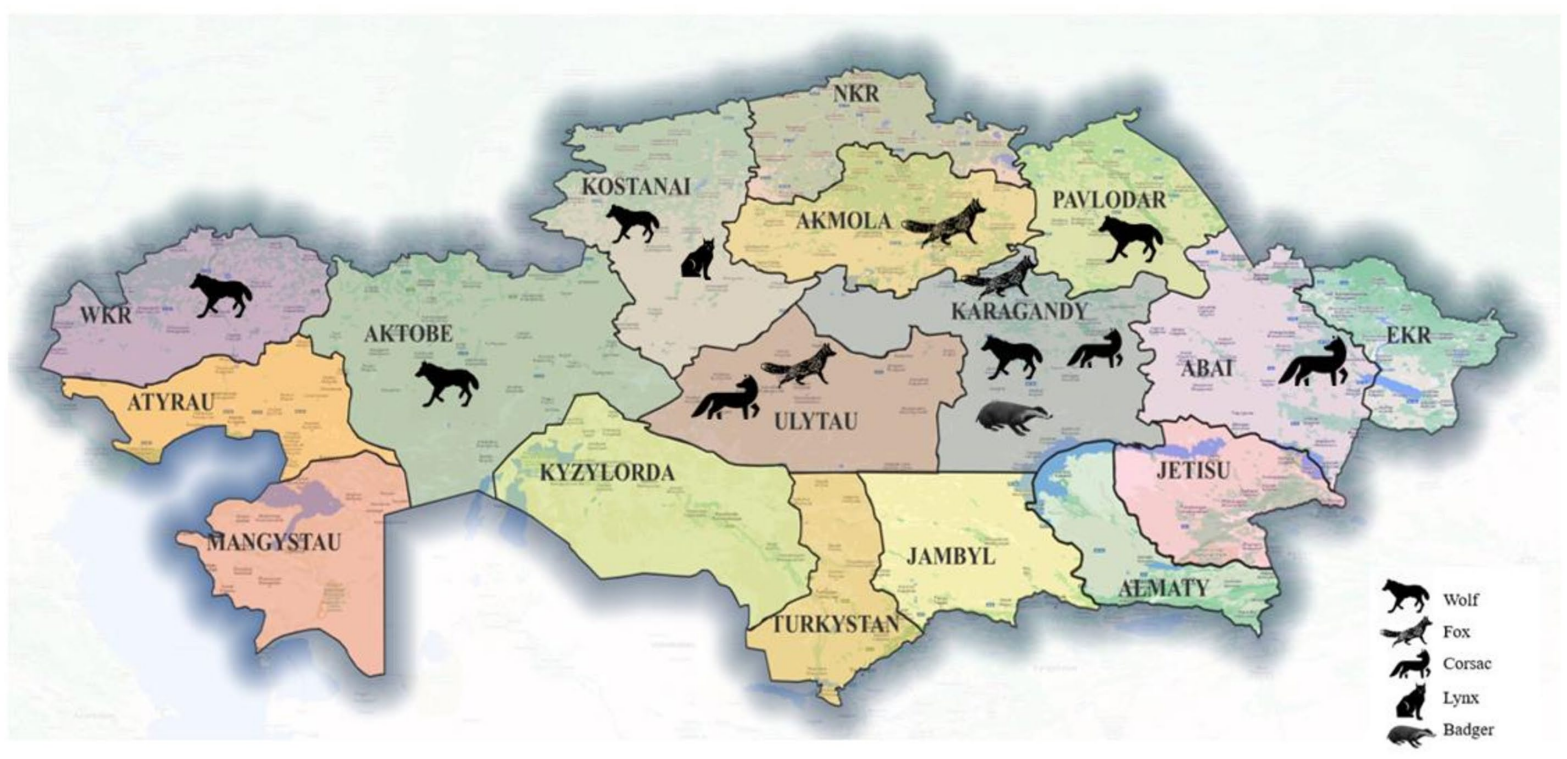
Map of Kazakhstan showing the provinces and the geographical origin of the host species collected.

Adult wild carnivores, including 83 gray wolves (*Canis lupus*), 53 red foxes (*Vulpes vulpes*), 11 corsac foxes (*Vulpes corsac*), 3 European lynx (*Lynx lynx*) and 4 badgers (*Meles meles*) were available for this study. They had been shot by hunters in different regions of Kazakhstan (Figure 1) between December 2019 and October 2023. The gastrointestinal tract of each animal, frozen until examination, was examined for helminths as described by Skrjabin (25). Adult roundworms were collected, washed in physiological saline, morphologically identified to species (26, 27) and preserved in 70% ethanol.

### 2.3 DNA extraction

Following morphological specification, one worm from each ascarid-positive animal was randomly selected for molecular analysis. A small piece of this specimen was cut off and homogenized, and the homogenate was subjected to the standard phenol-chloroform method supplemented with proteinase K, to extract genomic DNA (gDNA). The DNA was then precipitated with ethanol (28), purified, dissolved in ddH_2_O and stored at −70°C for subsequent analysis.

### 2.4 PCR analysis

First, a polymerase chain reaction (PCR) was performed using the universal NC13/NC2 primer pair to amplify worm gDNA (21). PCR was performed in a 25 μL reaction mixture containing 10× Taq buffer with (NH_4_)_2_SO_4_, 2.5 mM MgCl_2_, 1 U Taq DNA polymerase and 200 μM dNTPs (Thermo Scientific, Carlsbad, CA, USA), 10 pmol of each primer and 20 ng of extracted gDNA as a template. DNA segments were amplified using thermal cycling reactions for 30 cycles of denaturation (94°C for 30 s), annealing (55°C for 30 s) and extension (72°C for 30 s). The resulting amplification products were separated by electrophoresis on a 1.5% agarose gel prepared with 1× TAE buffer solution containing 8 ng/μL ethidium bromide. This was followed by species-specific PCR targeting the partial internal transcribed spacer 2 (ITS2) ribosomal DNA (rDNA) gene of *Toxocara canis*, *Toxocara cati* and *T. leonina* using the primer pairs Tcan1/NC2, Tcat1/NC2 and Tleo1/NC2, respectively, (21). All PCRs were performed as described by Jacobs et al. (21). For the identification of *Baylisascaris* sp. a primer pair targeting the ITS1-5.8S-ITS2 rDNA genes was used under the conditions described by Franssen et al. (29). The sequences of all primers used are shown in Table 1.

**Table 1:** List of primers used in this study.

Parasite	Target gene	Primer name	Primer sequence (5′–3′)	Reference
Universal nematode	5.8S	NC13	F: ATCGATGAAGAACGCAGC	(21)
		NC2	R: TTAGTTTCTTTTCCTCCGCT	
*Toxocara canis*	ITS2	Tcan1	F: AGTATGATGGGCGCGCCAAT	(21)
		NC2	R: TTAGTTTCTTTTCCTCCGCT	
*Toxocara cati*	ITS2	Tcat1	F: GGAGAAGTAAGATCGTGGCACGCGT	(21)
		NC2	R: TTAGTTTCTTTTCCTCCGCT	
*Toxascaris leonina*	ITS2	Tleo1	F: CGAACGCTCATATAACGGCATACTC	(21)
		NC2	R: TTAGTTTCTTTTCCTCCGCT	
*Baylisascaris* spp.	ITS1-5.8S-ITS2	ITS1-5.8S-IT2-F	F: ATAGTGAGTTGCACACTAATGT	(29)
		ITS1-5.8S-ITS2-R	R: TTATATGCTTAAATTCAGCGGG	

F, forward primer; R, reverse primer.

### 2.5 Sequencing analysis and phylogeny

Two positive amplification products were randomly selected from each host species for sequencing and genotyping. The respective amplicons were purified using a Quick PCR Purification Kit (Invitrogen, Lithuania) according to the manufacturer’s protocols. Sequencing was performed according to the Seq Studio Genetic Analyzer manual (Thermo Fisher Scientific Applied Biosystems, USA). The nucleotide sequences were visually checked using the Bio Capt program (version 11.0) and then analyzed by BLAST search against the GenBank database.^2^ Finally, the nucleotide sequences were aligned using the Clustal W program, and the relationships of the taxa were analyzed with 1,000 bootstrap replicates by the maximum likelihood method with MEGA11 (30). For the inference method, the nearest neighbor Interaction (NNI) was used. The tree for *Baylisascaris* species was rooted by the outgroup *Anisakis nascettii* (JX486104).

### 2.6 Statistical analysis

Explorative data analysis was performed using the BIAS statistical software (31). The observed prevalence, mean intensity and abundance of each ascarid species were calculated as described by Bush et al. (32).

## 3 Results

A total of 211 adult ascarids were collected from 154 host animals. Based on morphology, three species were identified: wolves (9.6% infected), red foxes (49.1%) and corsac foxes (55%) were infected only with *T. leonina*, lynx (66%) and badgers (50%) were infected only with *Toxocara cati* and *Baylisascaris* (*B.*) *melis*, respectively. Their mean intensity and abundance were low (Table 2). Adult *Toxocara canis* were not found in any of the hosts.

**Table 2:** Prevalence, intensity and abundance of adult ascarid species on the basis of morphology in wild carnivores in Kazakhstan.

Host	N infected/N examined	% prevalence (95% CI)	N worms found	Range of intensity	Mean (SD) intensity	Mean (SD) abundance	Ascarid species identified
Wolf	8/83	9.6 (4.3–18.1)	34	2–6	4.3 (1.3)	0.4 (1.3)	*Toxascaris leonina*
Red fox	26/53	49.1 (35.1–63.2)	134	2–8	5.1 (1.7)	2.5 (2.9)	*Toxascaris leonina*
Corsac fox	6/11	55 (23–83)	27	3–6	4.5 (1.0)	2.6 (2.5)	*Toxascaris leonina*
Lynx	2/3	66 (9–99)	7	2–5	3.5 (2.3)	2.1 (2.5)	*Toxocara cati*
Badger	2/4	50 (0.7–93)	9	2–7	4.5 (3.5)	2.3 (3.3)	*Baylisascaris melis*

95% CI, 95% confidence interval; SD, standard deviation.

**Figure 2:**
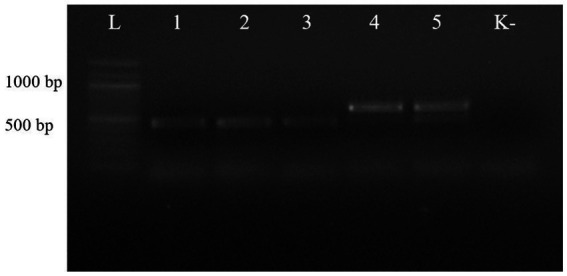
Electrophoresis of PCR products of gDNA from representative ascarid samples using the universal primer pair NC13/NC2: lane L: DNA marker; lanes 1–5: gDNA from ascarids collected from red fox (1), wolf (2), corsac fox (3), lynx (4) and badger (5); lane K: negative control (ddH_2_O).

The first PCR performed with the universal primer pair NC13/NC2 showed that the length of the PCR products from the ascarids of canids (wolf, red fox, and corsac fox) was different from that of the PCR products from the worms of lynx and badger (Figure 2).

**Figure 3:**
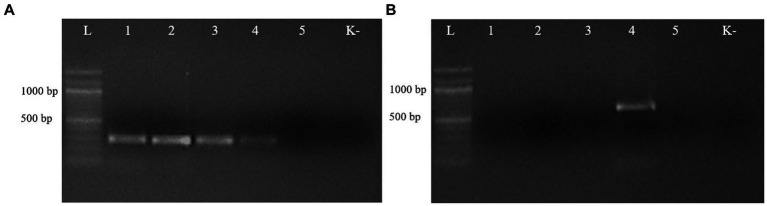
Electrophoresis of PCR products of gDNA from representative ascarid samples using the primer pairs Tleo1/NC2 **(A)** and Tcat1/NC2 **(B)**, species-specific for *Toxascaris leonina* and *Toxocara cati*, respectively. Lane L: DNA marker; lanes 1–5: DNA from ascarids collected from red fox (1), wolf (2), corsac fox (3), lynx (4) and badger (5); lane K: negative control (ddH_2_O).

The second PCR, performed with the respective species-specific primer pairs targeting the ITS2 rDNA region, identified *T. leonina* in canids and *Toxocara cati* in lynx (Figure 3). The primer pair specific for *Toxocara canis* gave no results in any sample (data not shown). Ribosomal ITS2 amplicons were obtained from six *T. leonina* isolates (232–261 bp), two each from wolf, red fox and corsac fox, and from two *Toxocara cati* isolates (375 and 434 bp) from lynx. The badger ascarids were identified as *Baylisascaris* sp. using a primer on the ribosomal ITS1-5.8S-ITS2 region and by comparison of the nucleotide sequences obtained with references from the GenBank database. Ribosomal ITS1-5.8S-ITS2 amplicons of 511 bp and 842 bp in length were obtained from two *B. melis* isolates. Nucleotide sequence data for all isolates have been deposited in the NCBI GenBank database under the accession numbers shown in Table 3.

**Table 3:** GenBank accession no. and number of nucleotide base pairs of representative samples of adult ascarids from this study.

Species	Host	Accession no.	N bp
*Toxascaris leonina*	Wolf	OR647588	261
	Wolf	OR647594	241
	Red fox	OR647692	242
	Red fox	OR647694	232
	Corsac fox	OR647689	234
	Corsac fox	OR647691	235
*Toxocara cati*	Lynx	OQ975261	434
	Lynx	OQ975262	375
*Baylisascaris melis*	Badger	PP333110	842
	Badger	PP333114	511

**Figure 4:**
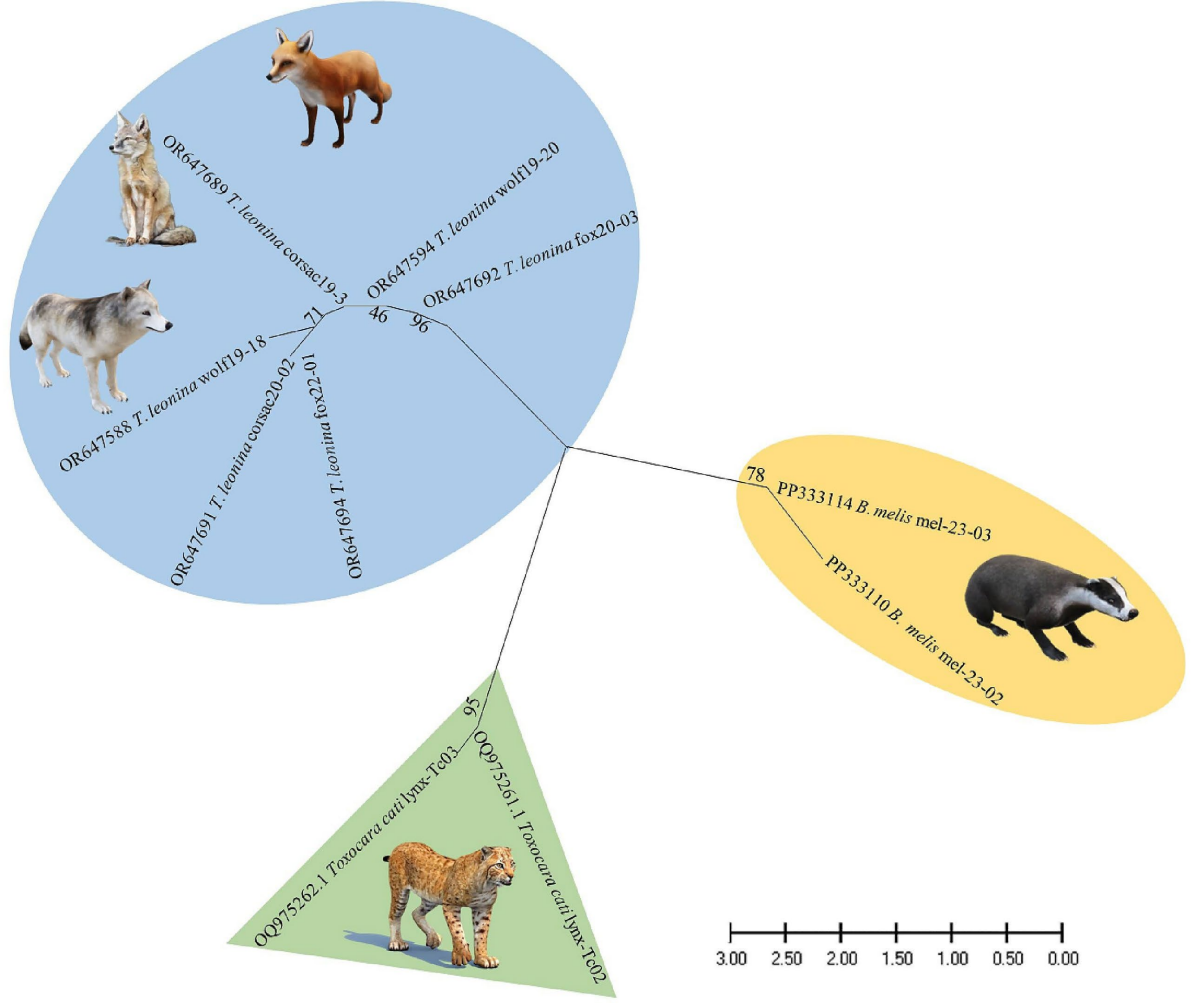
Maximum likelihood phylogeny constructed from nucleotide data of representative ascarid samples from wild carnivores in Kazakhstan. Numbers along the branches show bootstrap values resulting from different analyses in percent; scale: estimated number of nucleotide substitutions per site; species name and host species after the GenBank accession no.

**Figure 5:**
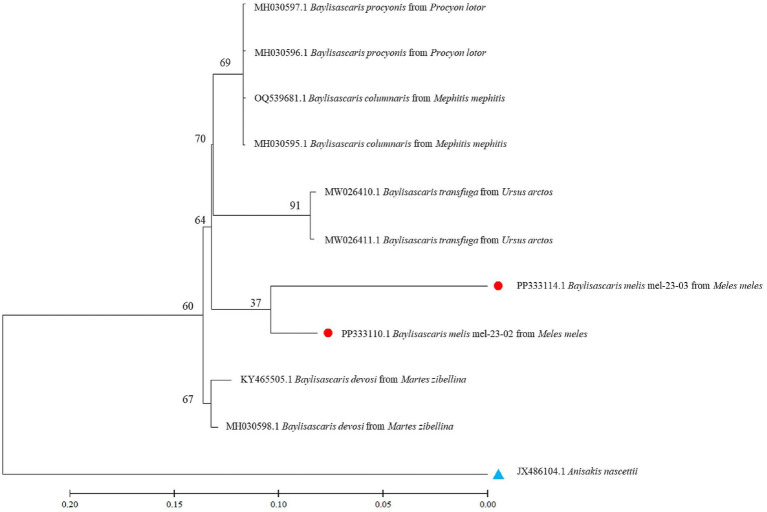
Maximum likelihood tree based on ribosomal ITS1-5.8S-ITS2 gene regions from several *Baylisascaris* spp. available in the GenBank and two isolates from this study. Numbers along the branches show bootstrap values resulting from different analyses in percent; scale: estimated number of nucleotide substitutions per site; species name and host species after the GenBank accession no.; red dots: isolates from this study; blue triangle: outgroup.

Nucleotide sequences from representative ascarid samples of the five host species were used to construct the maximum likelihood phylogenetic tree. Three distinct clades were identified: *T. leonina* from canids was placed in one clade with bootstrap values ranging from 46 to 96, *Toxocara cati* from lynx in another and *B. melis* from badgers in a third (Figure 4). Maximum tree analyses of the ribosomal ITS1-5.8S-ITS2 gene sequence showed that the two *B. melis* isolates formed a clade with the four reference species *Baylisascaris columnaris, Baylisascaris procyonis, Baylisascaris transfuga*, and *Baylisascaris devosi.* Both *B. melis* isolates showed slight genetic differences (Figure 5).

## 4 Discussion

In this study, five wild carnivore species in Kazakhstan were examined for their respective ascarid species. The species found, their prevalence, intensity and abundance partly differ from those of other Kazakh studies. This is not surprising as the regions of origin of the sampled hosts were different. It should also be noted that the data presented (as from previous studies) are not representative. They are based on a relatively small number of non-randomly selected hosts in a few regions of Kazakhstan, a large country of 2,725,000 km^2^, where, for example, the wolf and red fox populations are estimated to be 30,000 and 75,000, respectively, (33, 34). It is also well documented that the ascarid fauna of wild carnivores varies between landscapes (e.g., steppe, foothills, mountains) (19, 35, 36), which may be explained by local differences in prey availability (10). Furthermore, lynx are protected species and their killing requires justified exemptions. It is therefore quite difficult to study representative samples of these wild carnivores in such large countries.

Nevertheless, it is the first study to use molecular methods to identify ascarid nematodes from Central Asian countries. Phylogenetic analysis revealed three distinct species: *Toxocara cati*, *T. leonina*, and *B. melis* (Figure 4), confirming the morphological diagnosis.

In the three canid hosts, only *T. leonina* was identified, but not *Toxocara canis*. This is consistent with previous findings, based on traditional morphological methods, that *T. leonina* was the dominant ascarid species in corsac foxes in Kazakhstan (11), wild canids in southern Siberia (15), and stray dogs in Eurasian regions (37). It may be due to the higher cold tolerance of *T. leonina* eggs compared to *Toxocara canis* eggs, which favors this roundworm species in colder regions (37). However, it should be noted that the worms in the present study were obtained from adult hosts. This may have biased the results, as *Toxocara canis* is known to be mainly found in young canids (1, 26). In fact, other studies in Kazakhstan and neighboring countries have reported that wolves, red foxes or corsac foxes are infected with both ascarid species (12–14, 16–18).

*Toxocara cati* was the only ascarid species found in lynx. This was consistent with most reports from different countries (10, 15, 18), although occasionally *T. leonina* was also reported from this felid species (2, 38).

Wild canids and felids (as well as their domestic relatives) infected with ascarids contaminate the environment by excreting worm eggs in feces. The embryonated eggs are a potential source of infection for domestic dogs and cats and for paratenic hosts, including humans, who may ingest these eggs (1, 9, 10). In the light of the results presented, this infection risk can be assessed as follows: (a) *T. leonina* is considered a negligible parasite from a veterinary and zoonotic point of view (37). (b) In contrast, *Toxocara cati* is pathogenic in its definitive feline host (39) and also in paratenic hosts: Experimental studies have shown that after ingestion of *Toxocara cati* eggs, larvae migrate into tissues, including the brain, causing pathomorphological alterations in mice and pigs and abnormal neurobehaviour in mice (40, 41). It should therefore be considered as a potential cause of neural larval migrans symptoms in humans (42). However, lynx are likely to be a negligible source of *Toxocara cati* infection to humans, at least in Central Asia. This is because the lynx prefers to live in forested areas, which provide sufficient cover for hunting and abundant prey without much contact with human settlements (43). (c) *Toxocara canis* may be present in wolves and red foxes (see above), although not in this study. These wild canids are more synanthropic than the lynx, and their range extends close to human settlements (10, 44). This increases the risk of successful transmission of their parasites, including the zoonotic *Toxocara canis*, to domestic animals and humans (1, 9, 10).

This study also presents the first molecular data and provides the first phylogenetic analysis of *B. melis* worldwide. The badger ascarid was shown to be genetically distinct from *Baylisascaris* spp. of other carnivores: *B. columnaris* (definitive host: skunk [*Mephitis* spp.]), *B. procyonis* (raccoon [*Procyon lotor*]), *B. transfuga* (bears [*Ursus* spp.]) and *B. devosi* (marten [*Martes* spp.], fisher [*Pekania pennanti*], wolverine [*Gulo gulo*]) (Figure 5). This also confirms the morphological differentiation by Sprent (45) and supports the hypothesis (46) that the ascarids found in North American badgers (*Taxidea taxus*), which have been described as *B. columnaris*, are in fact *B. melis*. The significance of the slight genetic differences between the two *B. melis* isolates analyzed remains to be investigated. The phylogenetic analysis also showed that *B. procyonis* and *B. columnaris* form a clade. This confirms previous results suggesting that they are closely related species or that the former is even a synonym of the latter (47, 48).

Interestingly, there is little information on the geographical distribution and prevalence of *B. melis* in badger populations in Eurasia. First described over 100 years ago in Belgium (49), this is the second unequivocal identification of this species. This nematode had not been mentioned in any relevant study in central, western or southern European countries. There are two studies from Italy and Switzerland reporting only unspecified “ascarid” eggs or worms in a few badgers (Table 4). In contrast, ascarids have been collected from badgers in Uzbekistan, Azerbaijan and Caucasian Russia and morphologically identified as *Toxocara canis*, *B. columnaris* or *B. devosi* (Table 4). However, it is most likely that these worms were misidentified and were actually *B. melis*; the molecular results support this assumption. Thus, data from the literature and the results presented here suggest that *B. melis* may occur primarily, if not exclusively, in badger populations of western and central Asia. The reasons for this are still unknown.

**Table 4:** Results of previous studies on intestinal helminths, including ascarids, in badgers in Eurasia.

Country	N ascarid positive/N examined	Method used	Reference
Uzbekistan	0/19	Nec	(17)
	4/25 “*Toxocara canis*”	Nec	(18)
Azerbaijan	10/43 “*B. columnaris*”	Nec	(20)
	4/43 “*B. devosi*”		
Russia (Caucasus)	3/60 “*B. columnaris*”	Nec	(19)
Poland	0/17	Cop	(51)
Slovenia	0/18	Nec	(52)
Croatia	0/13	Nec	(53)
Austria	0/20	Nec	(54)
Germany	0/16	Nec	(55)
	0/84	Nec	(56)
Switzerland	2/249 “ascarids”	Nec	(57)
Italy	0/19	Nec	(58)
	1/43 “ascarid egg”	Cop	(59)
	0/18	Nec	(60)
Spain	0/85	Nec	(61)
	0/26	Nec	(62)
Portugal	0/163	Cop	(63)
Great Britain	0/118	Nec	(64)
Ireland	0/50	Cop	(65)
	0/289	Nec	(66)

Nec, necropsy; Cop, coproscopy.

It should be noted that *B. melis* is able to infect rodents (facultative intermediate hosts) under experimental conditions: It was highly pathogenic and caused fatal neural larva migrans symptoms in the American ground squirrel (*Urocitellus armatus*); mice (*Mus musculus*) did not develop clinical symptoms, but their brains and other tissues contained *B. melis* larvae (50). Whether this can also occur in Central Asian ground squirrel species (*Spermophilus* spp.) or other rodents under natural conditions does not seem impossible and requires further study. In any case, based on the clinical and pathological findings in rodents, a zoonotic significance of *B. melis* cannot be excluded and should be further investigated.

This study concludes by identifying ascarid nematodes from five distinct wild carnivore species in Central Asia within the phylogenetic framework. The study also presents the world’s first molecular data on *B. melis* from badger. It provides further insights into the classification and genetic diversity of ascarids. It reiterates the need for molecular methods to complement traditional morphological methods as a basic diagnostic tool in the future, for example in studies of the fauna, diversity, ecology and epidemiology of wildlife parasites, especially potential zoonotic agents. For future research, we are also considering collecting feces from wild carnivores to detect roundworm infection, which would increase the sample size.

## Data Availability

The datasets presented in this study can be found in online repositories. The names of the repository/repositories and accession number(s) can be found at: https://www.ncbi.nlm.nih.gov/nuccore/OR647588, OR647588; https://www.ncbi.nlm.nih.gov/nuccore/OR647594, OR647594; https://www.ncbi.nlm.nih.gov/nuccore/OR647692, OR647692; https://www.ncbi.nlm.nih.gov/nuccore/OR647694, OR647694; https://www.ncbi.nlm.nih.gov/nuccore/OR647689, OR647689; https://www.ncbi.nlm.nih.gov/nuccore/OR647691, OR647691; https://www.ncbi.nlm.nih.gov/nuccore/OQ975261, OQ975261; https://www.ncbi.nlm.nih.gov/nuccore/OQ975262, OQ975262; https://www.ncbi.nlm.nih.gov/nuccore/PP333110, PP333110; https://www.ncbi.nlm.nih.gov/nuccore/PP333114, PP333114.

## Footnotes

^1^ http://ec.europa.eu/environment/chemicals/lab_animals/legislation_en.htm

^2^ https://www.ncbi.nlm.nih.gov/
